# The burden of pediatric diarrhea: a cross-sectional study of incurred costs and perceptions of cost among Bolivian families

**DOI:** 10.1186/1471-2458-13-708

**Published:** 2013-08-02

**Authors:** Rachel M Burke, Paulina A Rebolledo, Sally R Embrey, Laura Danielle Wagner, Carter L Cowden, Fiona M Kelly, Emily R Smith, Volga Iñiguez, Juan S Leon

**Affiliations:** 1 Hubert Department of Global Health, Emory University, Rollins School of Public Health, Mailstop 1518-002-7BB, 1518 Clifton Road NE, Claudia N Rollins Bldg. 6050, Atlanta, GA 30322, USA; 2 Division of Infectious Diseases, Emory University School of Medicine, Atlanta, GA, USA; 3 Instituto de Biología Molecular y Biotecnología, Universidad Mayor de San Andrés, La Paz, Bolivia

**Keywords:** Societal costs, Economic burden, Diarrhea, Pediatrics, Health economics

## Abstract

**Background:**

Worldwide, acute gastroenteritis represents an enormous public health threat to children under five years of age, causing one billion episodes and 1.9 to 3.2 million deaths per year. In Bolivia, which has one of the lower GDPs in South America, an estimated 15% of under-five deaths are caused by diarrhea. Bolivian caregiver expenses related to diarrhea are believed to be minimal, as citizens benefit from universal health insurance for children under five. The goals of this report were to describe total incurred costs and cost burden associated with caregivers seeking treatment for pediatric gastroenteritis, and to quantify relationships among costs, cost burden, treatment setting, and perceptions of costs.

**Methods:**

From 2007 to 2009, researchers interviewed caregivers (n=1,107) of pediatric patients (<5 years of age) seeking treatment for diarrhea in sentinel hospitals participating in Bolivia’s diarrheal surveillance program across three main geographic regions. Data collected included demographics, clinical symptoms, direct costs (e.g. medication, consult fees) and indirect costs (e.g. lost wages).

**Results:**

Patient populations were similar across cities in terms of gender, duration of illness, and age, but familial income varied significantly (p<0.05) when stratified on appointment type. Direct, indirect, and total costs to families were significantly higher for inpatients as compared to outpatients of urban (p<0.001) and rural (p<0.05) residence. Consult fees and indirect costs made up a large proportion of total costs. Forty-five percent of patients’ families paid ≥1% of their annual household income for this single diarrheal episode. The perception that cost was affecting family finances was more frequent among those with higher actual cost burden.

**Conclusions:**

This study demonstrated that indirect costs due to acute pediatric diarrhea were a large component of total incurred familial costs. Additionally, familial costs associated with a single diarrheal episode affected the actual and perceived financial situation of a large number of caregivers. These data serve as a baseline for societal diarrheal costs before and immediately following the implementation of the rotavirus vaccine and highlight the serious economic importance of a diarrheal episode to Bolivian caregivers.

## Background

In the developing world, acute gastroenteritis presents an enormous public health threat to children under five years of age, with an incidence of one billion episodes and 1.9 to 3.2 million deaths per year [1]. Pediatric diarrhea incidence has been shown to be inversely related to socioeconomic status, with children in poverty much more vulnerable to acute diarrheal episodes [2].

Bolivia (per capita GDP of $4,700 [3]) is one of the lowest-ranking countries in the Americas region in terms of the Human Development Index (taking into account measures of health, education, and income) according to the United Nations Human Development Report [4] and suffers from high rates of diarrhea-related infant mortality. Out of every 1,000 live births, 54 children die before the age of five, with an estimated 15% of these deaths attributable to diarrheal illness [5]. The costs of these diarrheal episodes can have severe financial consequences in a setting where 15.6% of the population lives on less than $1.25 per day (2008 estimate) [5].

Acute gastroenteritis presents an economic burden to both healthcare systems and patient families [6-8]. Although there is a universal insurance program that benefits Bolivian children under the age of five, not all medications or diagnostic tests are covered. Further, if medications are out of stock at the hospital, the family may have to pay out-of-pocket at a non-hospital pharmacy. Thus, Bolivian patient families may still face substantial out-of-pocket expenses and productivity losses associated with pediatric gastroenteritis-related hospitalizations and outpatient visits. Direct (“out-of-pocket”) expenditures include all costs paid directly by the family, encompassing medical costs (such as medications, tests, or consult fees) as well as non-medical costs (such as transportation or extra diapers) [9]. Indirect costs are defined as the value of the time lost by a caregiver and their spouse from income-generating activities during the acute episode of diarrhea [10].

Incurred familial costs due to pediatric diarrheal episodes have been quantified in several different studies. In Kenya (GDP per capita US$1,800 [11]) and Kyrgyzstan (GDP per capita US$2,400 [11]), two countries with a relatively similar economic situation to Bolivia, the estimated average per-episode total familial costs (direct and indirect) ranged from US$19.86 (Kenya [12]) to US$47.90 (Kyrgyzstan [13]) for hospitalized children. In Vietnam (GDP per capita US$3,400 [14]) direct costs alone were found to amount to US$31.83 per case in one study [6]. Findings in somewhat wealthier countries included total incurred costs of US$215.88 [15] for hospitalized children in Mexico (GDP per capita US$14,800 [16]) and direct costs of US$12.89 [17] per case in Brazil (GDP per capita US$11,900 [18]).

In a low-resource setting like Bolivia, incurred costs may sometimes represent a large proportion of a family’s overall economy. This ratio, the total incurred costs for a single diarrheal episode to the annual family income, can be termed the “cost burden” [19]. While absolute costs associated with pediatric diarrhea have been quantified in various settings, few studies specifically examine the relative measure of cost burden as it relates to pediatric gastroenteritis. A review of the literature identified only two studies in low- and medium-income countries (LMIC) that addressed cost burden using a similar methodology as the present study. In a study set in India (GDP per capita US$3,700), Mendelssohn et al. found that direct costs incurred per diarrheal episode ranged from 2.2% to 5.8% of the household’s annual income [20]. In the aforementioned study in Kyrgyzstan, Flem et al. found that family-incurred costs (including direct and indirect) totaled 2.5% of the mean annual household income [13]. Several studies have shown that incurred familial costs can be difficult for families to pay, causing some (particularly the already-impoverished) to resort to borrowing money to cover these expenses [20,21]. In the aforementioned study in India, more than 80% of lower-income households reported borrowing money to cover direct costs, as compared to only 35.7% of higher-income households [20].

Factors that may affect familial direct and indirect costs include treatment setting (e.g. rural primary care facilities versus urban referral hospitals) and appointment type (outpatient versus inpatient), though limited studies have specifically sought to quantify the potential effects of these characteristics. In the above-discussed study by Mendelssohn et al., direct familial costs for hospitalized patients in India were found to be significantly higher at an urban referral hospital as compared to a rural community hospital (p<0.001); direct costs were also higher for outpatients, but not significantly so (p=0.06) [20]. However, the proportion of direct familial cost to annual family income for one episode of diarrhea was high in both urban (5.8%) and rural (2.2%) hospitals [20]. Two other studies conducted in China, one in rural areas and one in urban hospitals, also showed higher mean direct familial costs for diarrhea inpatients compared to diarrhea outpatients (statistical significance not reported) [20,22,23]. In three cities studied in Brazil, direct familial costs associated with diarrhea outpatient visits represented the largest proportion of overall direct familial costs associated with diarrheal episodes (including hospitalizations), likely reflecting the greater number of outpatient visits compared to hospitalizations [17].

Pediatric diarrhea can cause not only financial difficulties, but also emotional distress for caregivers: Parents may feel frustrated that they cannot help their sick child, frightened by the severity of the illness, and fatigued by caring for their child [24]. In one analysis from European countries, parents whose children were hospitalized for rotavirus-associated diarrhea reported higher levels of stress as compared to those whose children were seen in an outpatient or emergency room setting [25]. Perceptions of financial stress differed in the above two studies (both in developed settings) [24,25], but were not explicitly compared with actual incurred financial difficulty. A review of the literature did not find any studies directly comparing financial stress or perceived cost burden to actual incurred cost burden.

The aim of this report is to describe total incurred costs and cost burden associated with caregivers seeking treatment for pediatric gastroenteritis across six Bolivian hospitals. The first objective is to quantify these costs. The second objective is to better understand the relationships among costs, treatment setting, and appointment type. An additional objective is to understand the relationships among perceptions of cost and actual incurred cost burden. These results should further complement a study from 2011, where the impact of pediatric gastroenteritis to the entire Bolivian health care system was estimated, and helped provide the rationale for introduction of the rotavirus vaccination program in 2008 [26].

## Methods

### Study population

The data presented were collected through a collaboration between Emory University in the United States and Bolivia’s “Instituto de Biología Molecular y Biotecnología de la Universidad Mayor de San Andrés” (IBMB, Bolivia) between 2007 and 2009. Study participants were interviewed in hospitals and outpatient clinics in four cities in Bolivia: La Paz, El Alto, Cochabamba, and Santa Cruz. In all, six sentinel hospitals were included (Hospital Boliviano Holandés in El Alto, Hospital Materno Infantil and Hospital del Niño in La Paz, Centro Pediatria Albino R. Patiño and Hospital German Urquidi in Cochabamba, and Hospital Mario Ortiz Suarez in Santa Cruz). All six hospitals are sentinel sites located in the four major cities of Bolivia. These sites include both privately and publicly funded hospitals as well as pediatric and general hospitals, and all provide in- and out-patient care. All hospitals were located in primarily urban / peri-urban areas, but did draw some patients from rural areas or peri-urban areas as well.

Study participants were identified by nurses, doctors, and study staff, based on a primary diagnosis of acute diarrhea (inpatients) or treatment for acute diarrhea (outpatients). Diagnoses were confirmed by clinicians. Eligibility criteria were as follows: patient was a child of less than 5 years of age, with a primary diagnosis of acute, non-bloody diarrhea; parent / caregiver was at least 18 years of age. Parents / guardians were consented by study staff.

The total sample size of 1,107 was estimated according to the “Guidelines for Estimating the Economic Burden of Diarrheal Disease With Focus on Assessing the Costs of Rotavirus Diarrhea” [9]. To achieve a 10% precision and 0.5 coefficient of variation for each hospital, at least 49 records were collected from each hospital. From these 1,107, a total of 874 had complete demographic information and are included in Table 1. Of this population, 535 observations had complete cost data and sufficient sample size for analysis of cost burden. Given sample size considerations, only inpatient (N=250) and outpatient (N=285) visits were considered from the six hospitals. Due to inappropriate sample sizes for analyses (< 10 children), for hospital German Urquidi, inpatient visits were excluded, and for Hospitals Materno Infantil and Del Niño, outpatient visits were excluded.

**Table 1:** Characteristics of the study population, by city

	**City**				
	**All cities**	**Cochabamba**	**El Alto**	**La Paz**	**Santa Cruz**
	**(n=874)**	**(n=402)**	**(n=112)**	**(n=146)**	**(n=214)**
	*Frequency (%)*				
**MALE**	493	222	64	86	121
	*(56.4)*	*(55.2)*	*(57.4)*	*(58.9)*	*(56.5)*
**URBAN RESIDENCE**^**1**^	433	267	-	-	137
	*(82.8)*	*(81.9)*			*(82.0)*
**HOSPITALIZED**	548	186	82	145	79
	*(62.7)*	*(46.3)*	*(73.2)*	*(99.3)*	*(36.9)*
**OUTPATIENTS**	326	216	30	1	135
	*(37.3)*	*(53.7)*	*(26.8)*	*(0.7)*	*(63.1)*
**TOTAL CHILDREN **^**2**^	874	402	112	146	214
		*(46.0)*	*(12.8)*	*(16.7)*	*(24.5)*
	*Median (25%, 75%)*				
**HOSPITALIZED**^**2,3**^					
Number of days child had diarrhea prior to visit	3.0	3.0	3.0	4.0	3.0
	*(2.0, 5.0)*	*(1.0, 5.0)*	*(2.0, 6.0)*	*(3.0, 6.0)*	*(2.0, 5.0)*
Patient’s age (months)	11.0	9.0	11.0	11.0	11.0
	*(6.0, 15.0)*	*(6.0, 15.0)*	*(8.0, 15.0)*	*(8.0, 16.0)*	*(6.0, 16.0)*
Monthly household income (US$)	152.06	151.20	115.20	144.00	172.80
	*(86.40, 288.00)*	*(60.48, 288.00)*	*(76.32, 216.00)*	*(86.40, 273.60)*	*(100.80, 259.20)*
**OUTPATIENTS**^**2,3**^					
Number of days child had diarrhea prior to visit	3.0	3.0	3.0	*-*	3.0
	*(2.0, 5.0)*	*(2.0, 5.0)*	*(2.0, 4.0)*		*(2.0, 4.0)*
	11.0	11.0	8.0	*-*	11.5
Patient’s age (months)	*(7.0, 17.0)*	*(7.0, 17.0)*	*(4.0, 13.0)*		*(5.0, 22.0)*
	172.80	151.20^†^	144.00^†^	*-*	259.20
Monthly household income (US$)	*(100.80, 288.00)*	*(86.40, 288.00)*	*(115.20, 216.00)*		*(144.00, 345.60)*

^1^There were substantial missing data for urban vs. rural residence—40.2% of observations were missing this information. Data from El Alto and La Paz are excluded from this comparison due to near-complete missing information.

^2^Percentages are calculated out of the total hospitalized and outpatient sample size.

^3^The inpatient appointment Hospital German Urquidi and the outpatient appointment Hospital Del Nino and Hospital Materno-Infantil were not analyzed due to insufficient data.

^†^Significantly different from Santa Cruz (p<0.01), using Kruskal-Wallis test followed by post-hoc pairwise comparison.

### Ethical approval

Prior to data collection, all portions of the study, including inpatient and outpatient chart abstraction, were approved by Emory University’s Institutional Review and the Bolivian National Bioethics Committee.

### Data collection

The costs of treating diarrhea were determined by conducting surveys with parents of sick children. The survey was developed based on guidance from the World Health Organization document “Guidelines for Estimating the Economic Burden of Diarrheal Disease with Focus on Assessing the Costs of Rotavirus Diarrhea” [9]. Parents and guardians of inpatient children were surveyed by researchers within a week of hospitalization. Parents and guardians of outpatient children were surveyed by study investigators while waiting for the outpatient appointment. Data were collected primarily during the winter months in Bolivia (May – September), given the seasonality of acute diarrheal illness in this population.

### Data management

All data were recorded by study investigators and stored in Epi-Info version 3.5.2. Two independent researchers entered the same data twice, data discrepancies were identified and documented, and discrepancies were resolved by consulting the original survey. Data were cleaned in SAS version 9.3 (Cary, NC).

Direct medical costs were defined as the costs of the consultation, drugs, tests, and any previous treatment for the same episode of diarrhea. Direct non-medical costs were defined as the cost of food during hospitalization or when waiting for the consultation, transportation from home to hospital, and caring for children when parents were in the hospital (including diapers and food). Indirect costs were defined as lost productivity of parents and guardians, determined using a function of salary and time missed from work (both caregiver-reported) due to the episode of diarrhea. Monthly family income was calculated from a function of daily, weekly, or monthly salary, and days worked per month as reported by the parents. The incomes of both parents were summed to create the total family income. The cost burden incurred by the families due to one episode of diarrhea was defined as the total direct and indirect costs incurred by the family divided by their annual household income and expressed as a percentage. Costs in Bolivianos (BOB) were converted into USD using the second quarter 2011 exchange rate of 1 BOB equal to US$0.144. Perceptions of the impact of incurred costs were assessed on a five-point Likert scale response to the question “Do you feel that paying for the treatment of your child’s diarrhea has affected your family finances? How much?” Responses of 4 (“More or less”) and 5 (“A lot”) were coded as “Yes,” while all other responses were coded as “No.” Data were also collected on the presence or absence of complications and comorbidities such as dehydration, vomiting, hypokalemia, metabolic acidosis, electrolyte disorders, malnutrition, anemia, bronchopneumonia, and acute respiratory infection, or another unspecified comorbidity or complication.

### Statistical analysis

Statistical analysis was completed using SAS version 9.3. Chi-squared tests were used to compare two proportions. Because cost data were not normally distributed, non-parametric tests were used for all cost analyses. When comparing medians between two groups, the Wilcoxon-Mann Whitney test was used. When comparing medians among multiple groups, the Kruskal-Wallis test was used, followed by post-hoc pairwise comparisons. The Cochran-Armitage test for trend was used to assess trend of cost perception by levels of cost burden. P values less than 0.05 were considered significant.

## Results

The primary aim of this study was to describe direct and indirect costs and the cost burden (total direct and indirect costs incurred by the family, as a percentage of their annual household income) associated with caregivers seeking treatment for pediatric gastroenteritis across six different Bolivian healthcare settings.

Among 548 inpatient surveys and 326 outpatient surveys with complete data on child demographics and location, the gender distribution among patients was approximately equal among the different cities, and within this population, 56% were male patients (Table 1). There were no significant differences among cities in the duration of diarrheal episode or patient age. The median monthly income was not significantly different between families of inpatient and outpatient children. However, within outpatients, familial income was significantly higher in Santa Cruz as compared to Cochabamba (p<0.025) and El Alto (p<0.002) (Table 1). In summary, only familial income was significantly different between different cities when stratified on appointment type.

Given that costs can also be different by urban as compared to rural residence, we also examined the distribution of demographics by residence location (Table 2). Overall, approximately 83% of participants reported urban residence. Characteristics were similar between the two populations, with the exception of number of days ill prior to present treatment, which was significantly higher for rural participants.

**Table 2:** **Characteristics of the study population, by rural / urban residence**^**1**^

	**Residence**		
	**All children**	**Urban**	**Rural**
	*Frequency (%)*		
**MALE**	292	250	42
	*(55.8)*	*(57.7)*	*(46.7)*
**HOSPITALIZED**^**2**^	203	167	36
	*(38.8)*	*(38.6)*	*(40.0)*
**OUTPATIENTS**^**2**^	320	266	54
	*(61.2)*	*(61.43)*	*(60.0)*
**ALL CHILDREN**	523	433	90
		*(82.8)*	*(17.2)*
			
	*Median (25%, 75%)*		
**HOSPITALIZED**			
Number of days child had diarrhea prior to visit	3.0	3.0	3.5
	*(2.0, 5.0)*	*(2.0, 5.0)*	*(2.0, 5.0)*
Patient’s age (months)	10.0	10.0	7.0
	*(5.0, 15.0)*	*(6.0,15.0)*	*(3.0, 15.0)*
Monthly household income (US$)	172.80	172.80	139.68
	*(93.60, 288.00)*	*(93.60, 266.40)*	*(86.40, 291.60)*
**OUTPATIENTS**			
Number of days child had diarrhea prior to visit	**3.0**	**3.0**	**4.0**
	***(2.0, 5.0)***	***(2.0, 5.0)***	***(2.0, 7.0)***
Patient’s age (months)	11.0	10.5	12.0
	*(7.0, 17.0)*	*(7.0, 17.0)*	*(7.0, 198.0)*
Monthly household income (US$)	172.80	172.80	172.80
	*(100.80, 288.00)*	*(113.76, 288.00)*	*(7.20, 288.00)*

^1^Results should be interpreted with caution due to large amount of missing data (40.2%) on rural / urban residence status.

^2^Percentages are calculated out of the column sample size.

^3^Bolded numbers are significantly different by a Kruskal-Wallis test at an alpha level of 0.05.

Since comorbidities can increase the cost of treatment, data were also collected on the child’s comorbidities and presenting complications. Of those with complete data (623 of 971), 40% of children presented with vomiting, 65% with dehydration, 10% with malnutrition, 9% with anemia, 6% with acute respiratory infection or bronchopneumonia, 14% with some electrolyte imbalance, and 13% with some other unspecified complication. Overall, 53% of study participants presented with some type of comorbidity or complication. Median total incurred costs were significantly higher (p<0.0001) among those presenting with at least one comorbidity or complication, as compared to those presenting with none (US$42 vs. US$26) (data not shown).

To understand potential correlates of caregiver costs, direct, indirect, and total incurred costs were stratified by type of residence and appointment (Table 3). This analysis was limited to 535 patients (250 inpatients and 285 outpatients) with complete cost burden data. Direct and indirect costs were significantly higher for inpatient care than outpatient care. Overall, total incurred costs were also significantly higher for inpatients than for outpatients. When total incurred costs for the entire study population were broken down by type of expenditure, consult fees totaled 30% of the incurred costs, indirect costs accounted for another 29%, and direct non-medical costs constituted 21%. This result indicates that consult fees and indirect costs, compared to other costs due to diarrheal episodes, were a large relative burden upon families and caregivers.

**Table 3:** **Caregiver costs incurred for a single episode of pediatric diarrhea (US$)**^1^

	**Urban**		**Rural**	
	**Hospitalized (N=132)**	**Outpatients (N=230)**	**Hospitalized (N=31)**	**Outpatients (N=49)**
	*Median (Q1, Q3)*			
**DIRECT COSTS**	27.58^†^	4.79	26.50^‡^	6.91
	*(5.98, 57.89)*	*(1.87, 14.54)*	*(9.07, 67.68)*	*(3.6, 18.72)*
**Diagnostics**	0.00	0.00	0.00	0.00
	*(0.00, 0.00)*	*(0.00, 0.00)*	*(0.00, 0.00)*	*(0.00, 0.00)*
**Medications**	6.48^†^	0.00	5.76	0.00
	*(0.00, 11.81)*	*(0.00, 5.04)*	*(0.00, 12.96)*	*(0.00, 5.83)*
**Consultation Fees**	7.20^†^	0.00	3.60^‡^	0.00
	*(0.00, 36.00)*	*(0.00, 0.00)*	*(0.00, 46.08)*	*(0.00, 0.00)*
**Non-Medical Costs (e.g., Travel, child care)**	4.03^†^	2.88	6.34	4.48
	*(2.63, 6.30)*	*(1.30, 5.62)*	*(3.31, 9.36)*	*(2.74, 9.50)*
**INDIRECT COSTS (lost income)**	12.00^†^	1.23	20.34^‡^	8.64
	*(0.00, 28.50)*	*(0.00, 13.37)*	*(11.52, 27.60)*	*(0.00, 24.48)*
**TOTAL COSTS**	41.54^†^	10.74	32.40^‡^	17.63
	*(10.7, 82.19)*	*(2.45, 29.95)*	*(15.7, 86.64)*	*(4.97, 36.91)*

^1^Only children with complete information on appointment type, hospital, and cost records were analyzed.

^†^Significantly different from Urban Outpatients, p<0.001.

^‡^Significantly different from Rural Outpatients, p<0.05.

**Figure 1:**
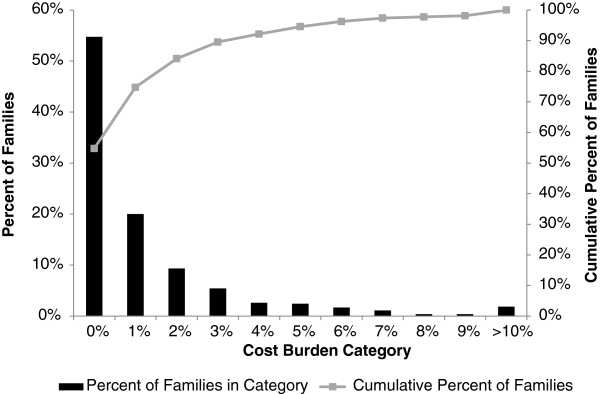
**Breakdown of cost burden categories.** Over 40% of Bolivian families in this study spent at least 1% of their annual income on a single episode of pediatric diarrhea. The black bars represent percentage of patients in each cost burden category. The gray line shows the cumulative percentage of patients in or below the current cost burden category. Each cost burden category, on the X axis, represents a range of one percentage point, for example, 0%=0 - <1%, 1%=1% - <2%. Only pediatric visits with complete cost records and hospital data were analyzed (N=535).

To assess the seriousness of an episode of pediatric gastroenteritis to the Bolivian family, the distribution of the cost burden was analyzed (Figure 1). Cost burdens ranged from less than 1% up to 38.17% (median 0.83%, IQR 1.83%), with 10 patients (1.9%) showing a cost burden of at least 10%. Though a majority (54.8%) of patients’ families experienced a cost burden of less than 1%, 45% of the patients’ families had to pay at least 1% of their annual household income for this single episode of diarrhea. For a family with the median annual income of US$1,749 (data not shown), a cost burden of 1% would translate into US$17.49 spent on a single episode of diarrhea. This could represent between three and five days of food and fuel for an average family of four persons, depending on the level of food security of the family [27]. Viewed through another lens, this amount is twice as much as the average monthly expenditure on intra-city transportation reported for Bolivians of “indigent” poverty in 2008 [28]. Inpatients (59.4%), compared to outpatients (33.7%), had a significantly higher frequency of paying more than 1% of their annual household income for this single episode of diarrhea and had a significantly higher median cost burden as well (1.36% inpatient versus 0.60% outpatient).

Additionally, relationships between actual incurred costs and perceptions of costs were explored. Overall, 68.5% of caregivers felt that diarrheal costs were affecting their family finances. The proportion of respondents reporting that cost affected family finances (4–5 on a Likert scale of 1–5) was significantly higher within higher levels of actual cost burden (p<0.001 for trend), with only 54% of those with a cost burden between 0 and 1% believing that the cost substantially affected family finances, as compared to 85% of those with a cost burden of at least 3%.

## Discussion

The objective of the present study was to describe the direct and indirect costs and the cost burden associated with caregivers seeking treatment for pediatric gastroenteritis at inpatient and outpatient settings in Bolivia. Families in this study incurred substantial costs for both inpatient and outpatient visits associated with pediatric diarrhea. Inpatient appointments were significantly more expensive than outpatient visits when considering indirect, direct, and total incurred costs. Additionally, indirect costs due to diarrheal episodes, though likely underestimated in the present study, were shown to be a large burden upon families and caregivers. Overall, most families felt that the diarrheal episode had a negative impact on their financial situation. These results demonstrate the high cost of inpatient care and the importance of indirect costs in an overall view of incurred familial costs associated with pediatric diarrhea.

This study demonstrated that the total incurred costs due to diarrheal episodes for inpatients were significantly higher than those associated with outpatient visits (Table 3). These costs are not negligible: total costs for a single episode of diarrhea in outpatients (median US$11.66) may represent between two and three days of food and fuel for a family of four, while those for hospitalized patients (median US$23.44) may represent between four and six days of food and fuel for a family of four, depending on the family’s level of food security [27]. Findings of greater costs for hospitalized patients were consistent with other studies of diarrheal costs in India and China, which also demonstrated higher familial incurred costs for inpatients as compared to outpatients [20,22,23]. Inpatient episodes (hospitalization) could result in higher direct and indirect costs due to required longer hospital stays, higher medical fees, and a greater need for the caregiver to take time off from work. Also consistent with studies in Vietnam [6] and India [20], indirect and nonmedical direct costs in our study were generally higher for rural patients as compared to urban patients, with higher rural indirect costs primarily seen in the ambulatory setting. This could be explained by a greater distance between the family’s home and the hospital or clinic, leading to higher transportation costs and a greater need for caregivers to take time off from work. Indeed, travel costs were significantly higher among rural families in our study, and the number of days the child had been ill prior to seeking the current treatment were also significantly longer (indicating a potential delay of treatment). Overall, data indicate that both direct and indirect costs are burdensome for Bolivian families of pediatric diarrhea patients, with the burden particularly high for rural families and those whose children are hospitalized.

A majority of patients had a cost burden of less than 1%. Cost burden estimates in similar studies ranged from 2.2% to 5.8% in India and up to 37% in Uzbekistan [20,29]. In this study, cost burden was generally higher for inpatients as compared to outpatients, reflecting the overall greater cost associated with hospitalization in Bolivia [26]. Though the cost burden of a single diarrheal episode was minimal for many caretakers in this study, nearly 45% of surveyed families spent at least 1% of their income on just one diarrheal episode. In this study, the 1.9% of patients experiencing cost burdens of at least 10% were found to have a range of familial incomes, from very low (US$7.20 per month) up to slightly above the median income within the study population (US$158.40 per month), demonstrating that families from a variety of income levels can experience a tremendous burden from even a single diarrheal episode. On the whole, this study population would be considered quite poor even within Bolivia: the median per capita income (US$45.43 per month) in the study population is nearly identical to the “Indigent Poverty” per capita income for Bolivia as calculated in 2000 ($45.20 per month) [4]. This may reflect an overall disparity in rates of pediatric gastroenteritis among different income groups in Bolivia. When families are already on a small budget and may spend over 60% of income on food and fuel (leaving little extra for healthcare, education, and housing expenses) [27], the cumulative economic impact of multiple pediatric diarrheal episodes may devastate a household’s overall financial well-being.

The results of the present study demonstrate that in Bolivia, indirect costs, such as lost wages, constituted a considerable fraction of the overall costs to caregivers and families. In a cost-of-illness study from Brazil [30], indirect costs accounted for 20% of total costs for inpatients, and 72% of total costs for outpatients, which is consistent with our own estimates and those reported in 2007 for the Latin American region by Rheingans et al. [15]. While these findings are consistent with two other studies, conducted in British and Canadian children [31,32], several other studies examining family-incurred costs for pediatric diarrhea in LMIC did not find indirect costs to make up a substantial proportion of overall costs [13,20,21,29]. There were several other differences in the methodology and study population between the present study and other studies on costs associated with pediatric diarrhea that may help to account for the distinct findings. While the other studies in LMIC used caregiver-reported daily wages and time lost from work to develop estimates of indirect costs (as in the present analysis) [13,20-23,29,33], studies with British and Canadian children used country mean daily wage values (and caregiver-reported time lost from work) to calculate indirect costs; further, parents in the British study did not incur any direct medical costs beyond over-the-counter medications [31,32]. In the studies where indirect costs were minimal, the authors mentioned difficulty in calculating average daily wages (e.g. because caregivers were employed informally and thus unable to estimate daily wages), as well as a low percentage of surveyed caregivers actually having missed work (because the caregiver who attended the sick child did not earn an income) as possible reasons for the low contribution of indirect costs. It’s possible that the present study found greater indirect costs due to the seemingly higher prevalence of income-generating activities by both spouses, as opposed to just one. Because the present study demonstrated that indirect costs were important to overall diarrhea-related costs in Bolivia, future economic studies and cost-effectiveness analyses focusing on societal costs in other countries should include indirect costs in their analyses.

This study also found that most families felt that the cost of the diarrheal episode had an impact on family finances. This is in contrast to a study on pediatric rotavirus infection in Canada, in which few families reported feeling that indirect costs (missing work) were impacting their family income [24]. However, most of these Canadian families were beneficiaries of paid absence or Family Medical Leave Allowance programs [24], while the Bolivian families in this study had no similar compensation for missing work. In addition, in the present study, the percentages of caregivers reporting that the episode affected them financially were higher as the actual cost burden increased. It’s possible that this reflects sensitivity on the part of the caregivers with regards to the real impact of cost burden. This finding suggests a need for research on caregiver costs to identify predictors of actual incurred cost and cost burden, which may in turn help to address the emotional stresses associated with this cost.

One strength of this study was the generalizability of its results to Bolivia because of the wide breadth of health care sites, covering multiple geographic regions of Bolivia and two types of health care settings (outpatient and inpatient). A second strength was the detail (i.e. multiple types of costs) with which data were collected. One limitation was that the cost estimates in this study are likely an underestimate of the true cost estimates because the caregiver sometimes was unable to provide specific cost information. A second limitation was that not all sites had sufficient sample sizes for a statistically meaningful comparison. Finally, the cross-sectional design of the study makes it challenging to examine the long term economic implications to the household resulting from death or other health consequences.

## Conclusion

In conclusion, this study has shown that indirect costs were a non-negligible component of a child’s diarrheal episode and that a single diarrheal episode affected the actual and perceived financial situation of a large number of caregivers. The Bolivian Rotavirus Surveillance Program estimates that, without vaccination, rotavirus alone causes over 47,000 outpatient visits, more than 9,000 hospitalizations, and 813 deaths each year [26]. Using data from the present paper, as well as a previously published cost-effectiveness analysis of the rotavirus vaccine in the Bolivian setting, we estimate that Bolivian families suffered pediatric diarrhea-related costs of US$2.8MM annually (range US$2.0MM to US$3.6MM), in this era prior to wide rotavirus vaccine implementation (data not shown) [26]. With the implementation of the rotavirus vaccine in Bolivia in 2008 [34-36], the number and severity of rotavirus diarrhea episodes, and therefore the societal costs due to diarrhea, are expected to decrease nationwide. The present data serve as a baseline for societal diarrheal costs before and immediately following the implementation of the rotavirus vaccine and highlight the serious economic importance of a diarrheal episode to Bolivian caregivers and the overall economic situation of the country, even in a setting with universal healthcare coverage for children.

## Abbreviations

LMIC: Low and middle-income countries.

## Competing interests

The authors declare that they have no competing interests.

## Authors’ contributions

RMB contributed data entry and data cleaning, performed statistical analysis, and drafted the manuscript. PAR contributed to the study design, data entry and statistical analysis, and in drafting the manuscript. SR.E contributed to the study design, performed data entry and data cleaning, and conducted initial statistical analysis. LDW, FMK, CLC, and ERS each contributed to the study concept and design and assisted in data collection. VI and JSL conceived of the study, participated in its design and coordination, and helped to finalize the manuscript. All study authors approved the final manuscript.

## Pre-publication history

The pre-publication history for this paper can be accessed here:

http://www.biomedcentral.com/1471-2458/13/708/prepub
